# “Alcohol will never run out”: Socio-ecological drivers of adolescent boys’ alcohol use in Kilimanjaro Region, Tanzania

**DOI:** 10.1371/journal.pgph.0002443

**Published:** 2024-06-10

**Authors:** Maaike L. Seekles, Winfrida Mwita, Alice Andongolile, Abdulrahaman Kihange, Gilbert Owen, Aliza Hudda, Blandina T. Mmbaga, Angela I. N. Obasi

**Affiliations:** 1 Department of International Public Health, Liverpool School of Tropical Medicine, Liverpool, United Kingdom; 2 Kilimanjaro Clinical Research Institute, Moshi, Tanzania; London School of Hygiene and Tropical Medicine Faculty of Epidemiology and Population Health, UNITED KINGDOM

## Abstract

Heavy alcohol use amongst adolescent boys is a major public health concern in many countries. It is associated with a range of negative physical and mental health outcomes and predicts alcohol-related problems in adulthood. In Kilimanjaro Region, adolescent boys’ alcohol use is widespread, and higher than other regions in Tanzania. An understanding of causal and contextual factors that influence the use of alcohol is needed to inform the development and implementation of effective alcohol prevention interventions. This study aimed to explore these socio-ecological factors in-depth amongst adolescent boys, young men and key stakeholders in Kilimanjaro Region, Tanzania. Between August 2022 and June 2023, multi-method, participatory, qualitative methods including: ethnographic observations (8 weeks), 37 in-depth interviews, 14 focus group discussions and participatory adolescent activities were used to elicit perceptions on factors driving adolescent boys’ alcohol use in two (rural/urban) settings. Data were triangulated and deductively analysed, guided by Bronfenbrenner’s socio-ecological framework. This study found many dynamic and inter-related factors linked to alcohol use within adolescents’ social, cultural, economic, regulatory, and physical environments. In a context of widespread availability of alcohol, low enforcement of alcohol regulation and (mis)conceptions around the benefits of alcohol use (e.g. curative and/or nutritional properties), parental and cultural influences largely determined the initiation of use in childhood and younger adolescence; employment status, peers, lack of alternative recreational activity and social norms around independence appeared to drive continued and increased use in older adolescence. Factors and their impact varied between rural and urban settings. In conclusion, a wide range of determinants and drivers of alcohol use among ABYM work at multiple socio-ecological levels especially parental, cultural and socioeconomic factors. This suggests that effective prevention requires a systems approach intervening across these levels. For example, incorporating education/awareness raising, increased law enforcement, parent-child communication and problem-solving, and income generation activities.

## Introduction

Harmful alcohol use among adolescents is a major public health challenge in sub-Saharan Africa (SSA) [1], where alcohol (beverages containing ethanol (ethyl alcohol) accounts for more deaths than in any other region [2]. In Tanzania, alcohol use disorders are twice, and rates of heavy episodic drinking are six times, the African Region average [2].

Adolescents are more vulnerable to alcohol-related harm per volume than adults [2]. Heavy alcohol use in adolescence is linked to several negative outcomes, such as impaired verbal learning and central nervous system development [3], mental ill-health (including self-harm and suicide) [4], sexual risk taking [5], intimate partner violence, and adverse HIV outcomes [2]. It is a key risk factor for road injury and interpersonal violence [6], which are leading causes of preventable deaths among adolescents in SSA [7]. Moreover, higher alcohol consumption in late adolescence predicts alcohol-related problems in adulthood [8].

Adolescence (age 10–19 years) is a crucial time to intervene, and particularly the reduction of alcohol use among adolescent boys and young men (ABYM) is a key priority. ABYM are more likely than girls to engage in alcohol use [2], and ABYM in Tanzania are at particular risk of use and excessive use of alcohol [9]. In the Kilimanjaro region specifically, Francis et al found that 64% of male secondary students in the area had ever used alcohol, often bottled beer or local brew, and 18% screened positive for alcohol use disorder. This was higher than other areas in the country [10]. Factors such as norms of masculinity and parental modelling have been implicated in increased alcohol use among boys and young men but understanding of these and other drivers is limited [10].

There is a need to delay and decrease alcohol use among ABYM, but interventions in SSA with proven efficacy to achieve this are lacking [11–13]. Key to designing such an intervention is an understanding of causal and contextual factors that influence the use of alcohol. This paper presents the findings of a multi-component qualitative and participatory study which aimed to explore these causal and contextual factors amongst ABYM in the Kilimanjaro Region, Northern-Tanzania.

## Methodology

### Theoretical framework

This investigation was guided by Bronfenbrenner’s socio-ecological framework [14], previously applied to an alcohol context by Sudhinaraset et al [15]. The framework posits that human health and development are affected by factors operating at multiple levels. In the context of alcohol use, individuals are nested within their individual microsystem (e.g. their home or work), which is nested within a mesosystem (a community) that is found in a macrosystem (a society) [15]. This paper reports the findings of individual, micro, and meso-level factors. A separate policy review, (Madundo et al; currently under review) has explored macro-level factors.

### Setting

The study included two sites: Ubetu, a rural village in Rombo District and Njoro, an urban ward of Moshi municipal, both in the Kilimanjaro Region, Tanzania. The villages were selected, after consultation with District officials and community organisations, as rural and urban settings with high rates of alcohol use and no prior existing interventions. In Ubetu, most community members are Christian and are part of the Chagga ethnic group, native to the Kilimanjaro region. People are largely dependent on agriculture (crop farming, poultry sale), or work as labourers in local timber industries or large-scale farms in neighbouring Kenya. In recent years, the economic situation in Rombo has worsened, as land has become less accessible. Conversely, the population in Njoro is largely Muslim and is not dominated by one ethnic group; inhabitants have migrated to the area from all over Tanzania and intertribal marriages are common. People mainly engage in day work and small business-related activities (e.g. as food vendors, hairdressers, alcohol sellers); however, there are slums and high levels of poverty in the area.

### Design

This study used a multi-method, participatory, qualitative approach. Ethnographic observations, in-depth interviews and focus groups with adolescent and adult community members, and participatory activities with in- and out- of school ABYM were conducted between August 2022 and June 2023 to investigate experiences, practices and perceptions related to the context and conditions of ABYM alcohol use. The study unit was the village. The adult and adolescent population formed the sampling frame from which interviewees and/or discussion participants were purposively selected, after introductory meetings and/or ethnographic observations (see below).

#### Ethnographic observations

For a period of 8 weeks, two male research assistants conducted ethnographic observations in each setting. The aim of these observations was to better understand the context, to observe (adolescent) drinking practices, and to build rapport with the communities to facilitate participant recruitment. Due to safety concerns, observations did not take place after 8pm. Research assistants used their phones and notepads to keep brief notes of observations and informal conversations, which they then typed up into detailed notes at the end of each day. Prior to observations, a one-day workshop took place with local level leaders and other community stakeholders to introduce the study, confirm that the study aligned with community problems and priorities, clarify study team intentions, and gain community level consent.

#### In-depth interviews and focus group discussions

Alongside observations, 37 semi-structured in-depth interviews and 14 focus group discussions (including 6–8 persons) were undertaken across the study sites. These included a wide range of purposively selected adult (e.g. teachers, health workers, alcohol brewers, religious leaders) and adolescent participants (see S1 Appendix for an overview of all participant characteristics). Topic guides were based on the socio-ecological framework and a-priori issues that are known to affect ABYM alcohol use at individual, micro and community levels. Any emerging themes were also explored in-depth. Data was collected by two male researchers, apart from the focus group discussions with AGYW, which were done by two female researchers. Prior to the interviews, participants were given a participant information sheet, which was also discussed verbally. Informed consent was obtained for all adult participants. For participants under 18, parental/guardian written consent was obtained, in addition to participants’ assent. All interviews/FGDs were held in Kiswahili (the local language) and captured on a voice-recorder.

#### Participatory workshop with ABYM

After initial analysis of interview data, a workshop was conducted with 16 in-and out-of-school ABYM (10–19 years) in which participatory activities were conducted to explore any gaps in researcher understanding and sense-check initial findings. The activities (described in Table 1) were adapted from an approach taken by Sommer and colleagues [16] and took place over 2 days. Parental/guardian written consent was obtained, in addition to participant assent. Activities were held in Kiswahili and translated in real time for the first author. The research team kept notes of discussions, activities were not audio recorded.

**Table 1:** Summary of participatory exercises conducted with ABYM in participatory workshop.

***Exercise 1*: *Types of alcohol and preferences*** Plenary game-based session where adolescents were passing around a ball–if they were given the ball, they would provide the name of an alcoholic drink. Two adolescents kept notes, to create a list of alcohol types. Based on the list, a discussion then took place around preferences and why certain types of alcohol would be consumed.
***Exercise 2*: *A day in the life of…*** Separated into 4 groups (Ubetu vs Njoro, in-school vs out-of school) ABYM were given pens and flipcharts and were asked to list the main activities, including timings, of a day in the life of the ‘average’ 12-year-old boy, 16-year-old boy in-school and 16-year-old boy out-of-school, in their respective communities. Once completed, they were asked to discuss the role of, or contact with, alcohol during these activities.
***Exercise 3*: *Prevalence of alcohol use*** As an individual exercise, ABYM were given a paper that contained six rows of 10 person icons. ABYM were asked to estimate and cross off, out of 10, the number of boys under 10, between 12–14 and between 15–17 years of age, how many would have ever and weekly drunk Mbege or Gongo in their respective communities.
***Exercise 4*: *Good & bad things about alcohol*** Separated into 4 groups, adolescents completed a table with a column for “good things about alcohol” and a column for “bad things about alcohol”, identifying what they feel belonged in each category.
***Exercise 5*: *Prioritisation of motivations of alcohol use*** In a plenary discussion, “good” things about alcohol in the previous exercise were summarised and framed as motivations for alcohol use. ABYM were then given post-it notes (a different colour for Ubetu and Njoro) and were asked to write down what they thought was the main motivation for a 12-year old adolescent and the main motivation for a 16-year old adolescent to drink alcohol.
***Exercise 6*: *Visual mapping of alcohol in community*** Participants were given flip-charts and pens, and asked to draw (and label) the places in their community where alcohol is sold or given out. The research team then discussed the map with the group to understand what types of alcohol are sold where, and any laws and regulations around the sale of alcohol in each venue.

### Data analysis

Field notes were read and summarised by the two researchers responsible for data collection. Recordings of interviews and FGDs were transcribed verbatim and translated by the research team. Quality of transcription and translation by the first researcher was independently checked by senior authors for all transcripts in the first phase of fieldwork. All subsequent transcripts were checked for any identified systematic errors. The transcripts and summary of observation notes were then imported into NVivo 12 software. The coding process largely followed a deductive approach. The first author and two other research team members developed a coding framework based on the socio-ecological framework and key concepts, categories and themes as identified in previous research on the topic. For quality assurance, the coding framework was iteratively assessed by three researchers who independently coded 4 interviews each and edited or inductively added new codes. Discrepancies were resolved by discussion until consensus was reached and a final coding framework was finalised. For further quality assurance 3 researchers independently coded all remaining transcripts and fieldnotes. Thematic analysis was used to explore the overarching themes and relationships between drivers of alcohol at different socio-ecological levels and across communities. Data from participatory activities were summarised and analysed using MS Excel (Ver 2311) were used to triangulate research findings and add further insight into prioritisation of factors driving alcohol use. Findings were fed back to community members during a workshop in June 2023; all agreed that the findings reflected a true and accurate representation of their context.

### Ethics and clearances

National and Institutional review board approvals were sought and granted from the local ethics committee of Kilimanjaro Christian Medical University College (Reference: 2551) and the National Health Research Ethics committee of the National Institute for Medical Research Coordinating committee in Tanzania (Reference: NIMR/HQ/R.8a/Vol. IX/3932) and the Liverpool School of Tropical Medicine in the United Kingdom (Reference: 21–075).

## Results

First, this section provides an overview of findings related to the types of alcohol that are consumed and ABYM drinking prevalence. Then it discusses the socio-ecological factors that were found to drive ABYM alcohol use, and highlights differences between study sites.

### Types of alcohol and drinking patterns

Findings from all data sources showed that there was a general distinction to be made between ‘local brews’ and ‘industrialised alcohol’. Local brews are made in the local area and are particularly prevalent in Ubetu. In Ubetu, alcohol is brewed in many homes (homebrews) and there are several small-scale industries that produce popular local beers called ‘Pombe ya Kijani’ (‘green alcohol’), named after the green bottles they are put in after production. Only a few of these are licensed. The main homebrewed drinks are Mbege and Gongo. Mbege, made from fermented bananas and millet, is the traditional Chagga drink and cheaply available. The alcohol content depends on the stage of the fermentation process and may thus vary for each batch; however, it generally contains a low percentage of alcohol. Mbege is mainly consumed for its cultural and perceived nutritional value, or to simply ‘quench one’s thirst’. The sale of Mbege is not regulated or taxed; it is openly consumed, often in a group context by sharing a mug. Gongo, on the other hand, is a strong spirit. The production and sale of Gongo is illegal and its consumption often more secretive. Indeed, during rapport building community concerns that the research team may be linked with, or report to, law enforcement agencies had to be addressed. In Njoro, there are also some locally produced drinks, such as Dadii (local brew) and Tangawizi (a licensed beer); Mbege and Gongo are generally imported for sale from nearby Chagga villages and therefore slightly more expensive. In Njoro, the consumption of ‘industrialised’ alcohol is more common than in Ubetu. These include licensed beers and other liquors from national and international brands. From ethnograhic observation liquors most commonly described as gins (Highlife, Konyagi, K-vant, and Diamond) were consumed in both settings and Kitoko, was more commonly consumed in Ubetu. Drinks described as brandy, (Valuer) and vodka (Azura) were also commonly consumed by ABYM in both settings. These liquors were generally sold at ~ 500 shillings (0.19USD) per glass.

Capturing the scope or prevalence of alcohol use amongst ABYM presented some challenges. First, many ABYM appeared to drink secretively, particularly if they are consuming drinks other than Mbege. They would get together in hiding spots (e.g. the forest) or conceal the alcohol they drink by putting it in energy drink bottles, which made it difficult for the study researchers to directly observe alcohol use amongst ABYM. Furthermore, questions around ABYM drinking patterns in interviews and FGDs received contradicting answers. This might be partly due to Mbege (which is also consumed by children and younger adolescents) not being considered alcohol by many community members. The general consensus, however, was that younger adolescents would drink more sporadically, whilst older ABYM have a more regular drinking pattern. Table 2 shows the results of the prevalence estimation exercise that was undertaken as part of the adolescent participatory activities workshop (Table 1, exercise 3). The exercise gave a rough quantitative estimate of ABYM perceptions of ever and regular alcohol use. The results suggest that almost every ABYM in Ubetu will have tasted Mbege at some point in their life. In Njoro, whilst the use of Mbege is slightly less, ABYM in that setting seem come into contact more with much stronger Gongo. It also suggests significant levels of alcohol use from a young age, although this differed by setting. For example, adolescents estimated that as many as 30% of those under 10 in Njoro will have ever tried Gongo, as opposed to 5% in Ubetu (Table 2).

**Table 2:** ABYM estimates of proportions of ABYM who have ever or regularly drunk Mbege or Gongo by age range and location.

	Estimated proportion of ABYM who have tried alcohol by alcohol type			
	Mbege		Gongo	
**Age range & location**	*Ever tried* Average out of 10 (range)	*Weekly use* Average out of 10 (range)	*Ever tried* Average out of 10 (range)	*Weekly use* Average out of 10 (range)
**Ubetu**				
<10 years	7 (5–10)	2.5 (2–3)	0.5 (0–1)	0.2 (0–1)
12–14 years	8.5 (7–10)	3.5 (4–7)	2 (0–3)	1 (0–2)
15–17 years	9 (8–10)	6.5 (5–9)	4 (3–5)	3 (1–5)
**Njoro**				
<10 years	4.5 (3–6)	2.5 (1–4)	3 (0–4)	1.5 (0–2)
12–14 years	6 (2–8)	3.5 (1–5)	5 (2–6)	2.5 (0–4)
15–17 years	7.5 (5–10)	5 (3–7)	6.5 (3–9)	4 (0–6)

### Factors and motivations for alcohol use

The following paragraphs present the identified socio-ecological factors related to ABYM alcohol use. During participatory adolescent activities, these factors were discussed and linked with identified motivations for drinking. An overview of all factors, combined with motivations, can be found in Table 3.

**Table 3:** Motivations for ABYM alcohol use linked to socio-ecological levels and factors.

Socio ecological level & Factors	Motivation for drinking alcohol
*Individual*	
Personality: Curiosity/Shyness/Dominance	• To explore new things/ to gain confidence
SES: Low (adversity, unemployment)	• To relieve stress • To have something to do/as a source of entertainment
SES: High	• To show status/money
Perceptions around outcomes of alcohol use	• For nutritional value/ to soothe hunger and thirst (mbege) • To cure illness • To gain energy/strength • To reduce physical pain • To reduce stress • To sleep better • To perform better sexually
*Microsystem*	
School and Employment environment	• To gain energy/strength/nutrition • To celebrate/reward for work achievement
Recreational opportunities	• Entertainment
Pressure from others • Peers • Adults	• To create strong friendship bonds • To fit in • To show that you are not weak
Parents/Family • Parent/child conflict • Parental drinking • Parental supervision	• To ease stress of parent-child conflicts • To try new things/imitation • Nutrition
Home environment • Alcohol brewed and sold in homes	• To try new things/nutrition/imitation • To test alcohol before selling/serving
*Community*	
Availability and access to alcohol	
Cultural/community norms	• To respect/maintain cultural identity • To fit in with the community
Social and gender norms	• To show independence/maturity

#### Individual level factors

*Personality*. Personality traits like curiosity and mirroring appeared to be linked to early initiation of alcohol use amongst younger ABYM. As natural human behaviours, experimentation and imitation are key elements of adolescence. Older ABYM explained that they consumed alcohol to increase confidence, either to reduce shyness or to increase the ability to assert one’s dominance. The relationship between these behaviours and alcohol use will be further discussed under sections related to microsystem and community factors.

*Socio-economic status*. Low socio-economic status due to unemployment, paired with boredom, was identified as a risk factor for excessive alcohol use, particularly for older ABYM. Participants in Ubetu and Njoro explained that those who were not in school and not in employment would form ‘gangs’ and engage in daily heavy alcohol use, to relieve stress, or to have a source of entertainment. In Njoro, these ‘gangs’ often pair alcohol consumption with drug use (mainly khat, marijuana and cocaine); in Ubetu, this appears less prevalent. Many participants from both communities mentioned that these ABYM would engage in petty theft (e.g. stealing chickens) to fund this lifestyle.

“*Too many…*.*the drinkers are very numerous*. *They are too many; you find a young person involved in bad gangs… smoking marijuana*, *drinking alcohol*…*–Out-of-school ABYM*, *Njoro*

“*He drops out of school; he is 13 or 12 years old*. *Now*, *when he is out of there (school)*, *he comes to the street and starts walking around*. *There is no employment around*. *When there is no work around*, *he indulges himself in drinking alcohol”*.*—Older ABYM*, *Ubetu*

“*The other thing is lack of employment*. *Once they don’t get employment they turn to alcohol use*, *but also to theft of things such as chickens and goats*, *which they sell so they can get money to buy alcohol*.*”–Father*, *Ubetu*

Particularly in Ubetu, the economic situation has worsened over the years. Fathers (and their sons) have reduced opportunities for income generation through farming, which has been linked to increased alcohol use amongst both:

“*Right now*, *the father doesn’t have a farm*, *and there is no place to take the child (to work)*, *so the father goes out to find a place to drink alcohol*, *and the child follows him there*, *and sometimes that’s where they meet*.*”–Father*, *Ubetu*

Conversely, ABYM with (relatively) high socio-economic status were said to consume and provide others with commercial alcohol as a symbol of relative wealth and success. In Ubetu, such behaviour was particularly displayed by adolescents who had moved to Kenya to find work, and who would return to the village during Easter, Summer holidays, and Christmas time.

*Perceptions/anticipated positive outcomes of consuming alcohol*. Several perceptions or misconceptions about the benefits of consuming alcohol were identified that provide insight into the motivations for alcohol use. There is the perception that Mbege provides nutritional value and can therefore be classed as food. Other, stronger types of alcohol are said to provide energy or physical strength needed for work, whilst food was perceived as making you lazy. In addition, alcohol is seen as a painkiller or is thought to have medicinal properties for curing colds and other respiratory illnesses such as COVID-19. Alcohol is further thought to reduce stress or negative thoughts, increase the ability to sleep and increase sexual urges. The links between these individual perceptions, broader micro-and community factors, and alcohol consumption will be discussed in more detail below.

“*People say that the alcohol that is made in our place is made from bananas and millet alone*, *there is nothing else intoxicating mixed in there*. *So*, *some parents consider it as just food*.*”–CHW*, *Ubetu*

“*Many say*: *‘Without drinking I can’t sleep well*. *The thoughts will choke me’*. *They drink so they can sleep*.*”–Salient person 2*, *Njoro*

#### Microsystem factors

*Parents and home environment*. Participants often referred to parents as the reasons for initiation of alcohol use.

“*The parents are the biggest influence when it comes to children’s alcohol use”*.*–CHW Njoro*

In both communities, examples were provided of how some parents are responsible for alcohol consumption amongst children from the moment they are born: Mbege is said to stimulate milk production in breastfeeding mothers and parents were said to give their children (including babies and toddlers) as a substitute/accompaniment for food, before they go to school, or whilst they are waiting for dinner to be ready. Stronger alcohols would be used to settle babies or make them sleep, or to help cure a cold.

“*You find children from primary school have come home from school and parents tell them to relieve their hunger with a little bit of Mbege while food is prepared*.*”–Teacher*, *Ubetu*

“*Others said that Gongo*, *or I don’t know*. *K-Vant*, *is children’s coughing medicine*…*you find she (mother) has just given it to the baby”*.*—AGYW*, *Njoro*

“*You may find that maybe the child is hungry*. *You find that the mother is already drunk*, *and you hear her saying ‘Why are you crying*? *Take this (alcohol) and drink’*.*”–Venue owner*, *Njoro*

Parental drinking, which children would imitate, was also identified as a risk factor. Children would taste alcohol if they were sent to buy it for their parents, or if it was available in the home environment (either for consumption or sale). Especially in Ubetu, children would often come into contact with alcohol through being involved in the production or sale of Mbege in their home. It was not uncommon for children to have to test the quality of the brew before selling it.

In both communities, ABYM would avoid parents finding out about their drinking by hanging out in ‘hiding spots’ and secretly consuming alcohol. In Njoro, parental monitoring was said to be very limited, including for younger ABYM. Many parents work away during the day, only to return late in the evening. Ethnographic observations found many children roaming the streets after school hours, and informal conversations indicated that ABYM often sleep over at friends’ houses without their parents’ consent. In Ubetu, parents were typically home in the evening, but parental supervision was mainly targeted at younger adolescents in primary school and girls.

Many parents expect independence of their sons after they leave primary school (standard 7), when ABYM are around 14 years–although some school-leavers are younger. At this point, parents may withdraw their (financial) support and expect ABYM to move out of the house. Familial tensions and arguments, because of alcohol use, were often mentioned in this context. Poor parent-child relationships, reduced parental monitoring and increased expectations of responsibility, paired with lack of economic opportunities (described above), appear to create an environment of stress and freedom that serves as a major driver for excessive alcohol use amongst older ABYM.

“*They drink regularly as they lack parental guidance*. *Parents don’t pay much attention to them*. *This is because*, *here*, *when an adolescent completes standard seven and joins secondary level*, *parents consider him as grown up enough*. *They leave him alone*.*” In-school ABYM*, *Ubetu*

“*If a child has completed the seventh grade*, *he and his mother do not get along at home*. *When his mother reprimands/criticizes him*, *he runs to alcohol*.”–*Out-of-school ABYM*, *Ubetu*

“*(They drink to relieve) depression from unemployment or loss of housing*. *If they make a mistake*, *or misbehave at home*, *parents will chase children out from their house*. *It is common*.*”–Out-of-school ABYM*, *Njoro*

“*Because when he gets drunk*, *he insults his parents*. *Now the parent makes the decision to chase him from home”*.*–Out-of-school AGYW*, *Ubetu*

*Influence and peer groups*. The initiation of alcohol use amongst children appeared to be mainly linked to adult influences; either from parents (discussed in previous paragraphs) or older adult community members.

“*It’s common among the Chagga community*. *That is*, *to find a child staying with grandmother or grandfather and the grandfather is brought alcohol and says: ‘I can’t drink without giving a child a taste’*. *You find that child isn’t 10 years old yet*, *even a child of 4 or 3 years old is just given a little to drink so that they sleep or rest (or so) that the old man who is drinking doesn’t feel like he is drinking alone*.*”–Community leader*, *Ubetu*

Sustained alcohol use of ABYM was linked to hanging out with ‘peer groups’. During social activities, boys and men from different ages would often mix, with those from older ages offering alcohol to younger ABYM. In the prioritisation exercise, ABYM (15–19 years) identified ‘building and strengthening friendships’, as a main motivation for alcohol use. Alcohol use was said to be unavoidable for ABYM who hang out with those who drink, as ABYM would ‘follow the trend’, ‘join in’, or ‘copy behaviour’. Social rejection, however, if you did not drink, was not specifically mentioned in interviews.

“*There are these adolescents*, *10 to 14 years old*, *they have friends who are already 18*, *or 20*. *They want to imitate and engage so easily in alcohol use*. *They call them ‘Nyokaa’*.*”–CHW*, *Njoro*.

“*At around the age of 14*, *when they reach puberty*, *they start using alcohol by imitating adults*, *their brothers*, *and relatives*, *based on the environment and what they see in their neighbourhood*.*”—ABYM*, *Njoro*

“*So*, *you can’t stay without drinking because you already have friends with whom you drink”*.*–Adolescent activity note*

*School-and work environment*. Whilst being in school or in full-time employment was identified as a protective factor against *excessive* alcohol use, the school- and work environments are not free from alcohol.

In both communities, younger adolescents (10–14 years) would generally attend school. The use of alcohol during school hours is strictly prohibited, and they would not generally come into contact with alcohol at school. However, a large proportion of the older ABYM in Ubetu (15–19 years) were said to leave the school premises to consume Mbege during lunch breaks. After school, in Ubetu, most ABYM would return home and complete chores (e.g. washing uniform, feeding animals). Some older ABYM in Njoro would also complete some chores for others (e.g. fetching water for neighbours, carrying bottles into a bar) to generate a small income. It is not uncommon for these ABYM to be given an alcoholic drink as a form of compensation for completing these chores.

ABYM in full-time employment would typically be involved in manual labour (e.g. timber industries in Rombo and garage workers in Njoro). In both communities, working ABYM were said to consume alcohol in moderate amounts during work hours, to gain energy and strength, and to reduce physical pain.

“*They think that alcohol stimulates their body*. *So*, *if they use it*, *they see it as an incentive for them to work*.*”–Older ABYM in school*, *Ubetu*

Alcohol may also be consumed in the work environment as a reward or as part of a celebration for the completed work. For example, in Njoro, boys working in the garage would have a beer after successfully repairing a car.

*Recreational activities*. There are few options for recreational activities in Ubetu; after school, few adolescents would meet at *vijiwenje* (meeting points) such as the playing ground or video venues, where they may consume alcohol, albeit secretively. In Njoro, there are more options for recreation (Play Station venue, football, swimming, pool table, drafts, video stores). Alcohol is not always sold at these venues, but may be brought in, concealed in soda bottles. Especially later in the evening, ABYM in school (both young and old) mix with adults at these venues and may consume alcohol. In both communities, the consumption of alcohol in itself was seen as a recreational activity. In Ubetu, it was said that most older adolescents might stop for some Mbege after work, but will then go home to sleep, whilst those in Njoro may meet their friends and have some alcoholic drinks together.

“*They can also meet around rivers*, *pretending to go for a swim while carrying their own drinks*, *they go there to drink*.*”–Teacher*, *Njoro*

“*They drink to water the heart (for pleasure or entertainment)”–CHW*, *Ubetu*

#### Community factors

*Community drinking culture*. In both communities, alcohol use (amongst men) is widespread, acceptable, and considered ‘normal’, although excessive consumption is frowned upon. In Ubetu, alcohol consumption is deeply rooted in culture; the Chagga traditionally celebrate, relax, commiserate, and socialise with Mbege in hand. The drink also has a social value, and is seen as a sign of prestige, generosity, or as a means to show respect or gratitude.

*If you give someone food*, *you are giving them charity*. *If you give someone alcohol*, *you show them respect*. *Note from adolescent activities*, *ABYM*, *Ubetu*.

Adhering to, or preserving cultural identity, was mentioned as one of the main motivations for early adolescent drinking. Traditional events, such as weddings, funerals, memorials, and celebrations around Christmas time, served as drivers for initial or sustained (albeit moderate) consumption. The cultural use of alcohol in Ubetu appears to be largely centred around communal consumption of Mbege or other, less strong, alcohol types. Alcohol is provided for free at these events. Adolescents are allowed to drink–and sometimes even expected to do so to show their respect. Another event that is often paired with alcohol consumption is the weekly meeting of a *Kiarano*, which is a support and micro savings group. Whilst the meeting is only for married adults, a bucket of Mbege is often set aside for adolescents who would be around.

“*Kiarano is a traditional activity*, *and alcohol must be present*. *Without alcohol people do not come to you*. *…*. *Yes*, *the child tastes it (alcohol) there*.*”–Teacher*, *Ubetu*

“*That is*, *there are two types of alcohol (at the Kiarano)*. *There is one for special people and there is one for young people*, *neighbours*.*”–Older ABYM*, *Ubetu*

“*It is completely acceptable that in traditional celebrations an adolescent can come and drink alcohol without anyone asking him or shouting at him because that is our custom here*.*”–In-school ABYM*, *Ubetu*

Two further specific examples showed how alcohol is engrained in cultural beliefs of the Chagga: Respondents explained that when a child is born, the parents would bury the baby’s umbilical cord and plant a banana tree on top of it. The bananas of this tree would then be used to brew Mbege, which is handed out to other community members. It is believed that the child will face adversity if this process is not followed. Furthermore, the Chagga often honour the deceased by passing on traditional names to their children. They believe that their child then carries the traits or characteristics of that person. A tendency for someone to overconsume alcohol was seen as a trait that could be passed on through names. Community members would explain and condone excessive drinking of an ABYM by saying that the person he was named after also drank a lot. In addition, giving alcohol to this ABYM would be like giving alcohol to that person, thereby respecting the ancestors.

Culture and tradition as drivers of alcohol use was less evident in Njoro. Widespread alcohol use was mainly linked adversity/poverty and a general environment of permissive neighbourhood norms around alcohol use that has created a ‘free space’ where people from nearby neighbourhoods in Moshi could go to drink without being judged. The use of strong alcohol, such as Gongo, appeared more common in this setting. There is a much higher population of Muslims in Njoro, and alcohol use is not generally central to events such as funerals and weddings. Religion, however, seems to have limited protective value within this context of widespread use.

“*Njoro is a centre*, *I think*. *It a place where many people come from other places*, *they come here to drink*, *for instance they come from town in Mbuyuni*, *or Majengo etc… it is a place where you can drink freely*.*”–CHW*, *Moshi*

*Social and gendered norms*. Drinking amongst older ABYM appeared to be closely linked to norms and perceptions around adulthood and the male gender. In line with the community drinking culture described above, ABYM and the wider community consider drinking as prototypical of adult male status. From school-leavers age, many ABYM consider themselves adults and start to drink more openly, to show their perceived independence and maturity. As discussed, many parents also expect independence of their sons from this age and would not monitor their son’s drinking. In addition, mothers in particular are often not able to reprimand their sons for drinking, due to gendered norms that encompass dominance of men over women. Gendered norms further stipulate that older ABYM/men are expected to socialise, instead of help with chores around the house, and socialisation comes with alcohol use. Strong social norms against female alcohol consumption and strict parental supervision of girls meant that alcohol consumption amongst AGYW was limited.

“*In other words*, *what causes it*, *I see*, *is the patriarchy*. *Because*, *a drunk girl will look like a freak*. *But if a male child is drunk*, *he doesn’t look weird*.*”—CHW*, *Rombo*

“*Those who drink more are boys*, *because they are considered as the father of the house… so they don’t look weird drinking*.*”—CHW*, *Ubetu*

“*They start drinking at 14 to 15 years*, *because they see themselves as adults and if they drink*, *the parents will not reprimand them”–Older ABYM*, *Njoro*.

*Physical and economic availability of alcohol*. The last, but not least, factor linked to alcohol use is ABYM’s ease of access to alcohol. In addition to the social availability of alcohol (communal drinking, free alcohol at events), physical and economic availability of alcohol is also high. The mapping exercise and interviews highlighted that both communities have a high alcohol outlet density, with multiple alcohol selling points in one street. Alcohol is sold in many homes and in small shops, in addition to more formal bars and clubs of different sizes.

“*Alcohol is easily accessible in our village; it is considered as any other commodity here*. *You may find that in a store*, *the stock of soda has run out*, *but the stock of alcohol will never run out”*.*—ABYM*, *Rombo*

“*I can even walk you around… among every ten to fifteen houses there is a bar*, *or a little shop*. *In that little shop*, *if you look closely*, *you find they sell apportioned (small measurements of) alcohol*.”–*CHW*, *Njoro*

Trading hours have been specified and in Ubetu a bell is rung every evening at 9pm to indicate that alcohol should no longer be sold. However, these hours are not observed and hard to enforce due to high outlet density and community members informing each other of impending police checks. In homes and shops, alcohol is said to be available at any time of day. Enforcement of opening hours of formal bars and clubs is more common, especially in Njoro, where the police have more of a presence than in Ubetu.

It appeared to be common knowledge that the law forbids sale of alcohol to those under 18 years of age. Yet, in both communities, alcohol was widely sold to minors, especially if they said that they were buying for their parents.

“*The environment to buy alcohol is easy*, *it is easy for a child to buy too*. *He just goes with money and gets the service*. *The service does not consider age*.*”—Religious leader*, *Njoro*

Furthermore, due to its very low price, the availability of alcohol is high. Most types of alcohol (especially the stronger, homebrewed ones) are sold in small servings, which means that individuals do not have to buy a whole (more expensive) bottle. Observations showed that the smallest serving of a drink may cost as little as 100 shillings ($0.04). It is also common for venues to allow ABYM to open a tab and pay later, which makes alcohol accessible at any time, even for those without a daily income.

“*Those which are put in bottles are sold very cheap*. *Even a form one or form two students (around 13/14 years old) can have it*, *he can afford it*.*”—CHW Njoro*

There were indications that due to the increasing price of bananas, the production and consumption of Mbege is reducing. However, globalisation has increased affordability and access to stronger, commercial alcohol. Informal conversations indicated that commercial alcohol used to be a luxury product, something that was only consumed on special occasions. Now, particularly in Njoro, its use is widespread.

## Discussion

This qualitative study found that alcohol use among ABYM is common. Consumption of the Local brew *Mbege* begins at an early age, and ABYM corroborate the views of older people that half or more of older ABYM use Mbege weekly. Alcohol use varied by location. ABYM reported that Mbege initiation was later and less common in Njoro vs Ubetu, whereas the pattern was reversed for Gongo, although Gongo initiation overall was less prevalent than Mbege.

### Factors determining alcohol use

The study used a social-ecological framework to explore how multiple levels of influence affect alcohol use amongst adolescent boys in an urban and rural setting in Tanzania’s Kilimanjaro region. The findings showed that alcohol use can be attributed to an interplay of a range of individual-, macrosystem-, and community-level factors.

Firstly, in both study sites, several factors play a significant role in predisposing ABYM to alcohol use. First, for cultural reasons and due to (mis)conceptions around positive outcomes of alcohol use, there is heavy alcohol consumption amongst adults. The most common misconceptions suggest alcohol has curative properties and nutritional or fortifying value. Second, limited enforcement of laws, and lack of recognition of home brewed liquors as alcoholic, further promote accessibility of alcohol for persons of all ages. Against this backdrop, compounding factors have differential effects on adolescents’ drinking behaviour. For example, early exposure to Mbege for family and cultural reasons may provide some protection against later habituation to stronger liquors. However, a general trajectory of alcohol use emerged. It confirmed findings of other studies that changes in adolescents’ social context and development lead to a shift in the weight of influence on their behaviour from the family unit during childhood, to peers during later adolescence.

Globally, parental alcohol use is often mentioned a factor that exerts great influence on young adolescents [17, 18]. Whilst this study concurs with these findings, it also identified influences from the family unit in this setting which go beyond that. Some children are introduced to alcohol (sometimes for nutritional benefit) from the moment they are born parents or other adult community members would give children alcohol, or children would have access to alcohol through involvement in alcohol production and sale at their homes. Similar practices have been reported in neighbouring Uganda and Kenya [19, 20]. Whilst this often involves limited consumption, it normalises alcohol use. As adolescents get older, alcohol use is more deliberate and is increasingly associated with socialisation with alcohol-drinking peers and broader social norms around adulthood. In the study settings, after adolescent boys leave primary school from the age of around 14 years, they are considered (and consider themselves) adults, rather than ‘older adolescents’. In line with broader community drinking behaviours, norms around male adulthood involve alcohol use. Moreover, there is an increased expectation of (financial) independence, which in a context of adversity and unemployment, for some, serves as a driver of alcohol use due to stress and parent-child conflict. The older adolescents ability to secure a stable income (or lack thereof), appears to largely define whether or not they are vulnerable to unemployment and a life centred around daily alcohol use. Although employment would often still involve alcohol use, albeit more moderately.

### Local socio-cultural variation

Whilst the above shows a general trajectory applicable to both study sites, there were also some important differences between the two areas. In Ubetu, alcohol use is heavily driven by cultural values of the Chagga people. The social consumption of homemade, unrecorded alcohol (Mbege) is an integral part of cultural gatherings (e.g. Kiarano) and ceremonies, such as weddings and funerals. Endorsing their cultural identity was one of the main drivers of alcohol use for ABYM. Yet, whilst these cultural values made ABYM in Ubetu vulnerable to early initiation and continued use of alcohol, the availability and expectancy of use of Mbege as a method for social interaction rather than intoxication, appeared to be a protective factor against the use of stronger industrialised or homebrewed alcohol such as Gongo. Cultural values appeared less influential in Njoro. Here ABYM alcohol consumption was influenced by the socio-economic environment and globalisation, which has led to increased availability and use of stronger, commercial alcohol at cheaper prices. ABYM combining alcohol with drug use (mainly cannabis, Khat and cocaine) in such settings is a concern that requires further investigation. Indications that this drug use is preceded by alcohol use further underline the need for preventive alcohol interventions in the area.

### Implications for intervention design

In terms of implications for the design of alcohol prevention interventions, our study found many dynamic and inter-related factors linked to alcohol use within adolescents’ social, cultural, economic, regulatory, and physical environments. In our view, prevention interventions in our study settings should respond by applying a systems approach to intervene across these multiple socio-ecological levels. Indeed, interventions based on education alone have shown limited effectiveness [21], and although evidence from SSA is limited, the WHO recommends multi-component community-based interventions (which include community mobilisation, enhanced community awareness and increased enforcement of laws) [2]. Whilst these are likely the most successful, in our study settings, they would also be essential to prevent children’s consumption of alcohol at an age at which they do not possess the knowledge or agency to refuse an alcoholic drink. Our findings also suggest that such an intervention should include income-generating activities and components related to parent-child communication and problem-solving, in order to address the challenges driving older ABYM alcohol use. A community-based participatory intervention design and implementation process would be essential to ensure cultural sensitivity of interventions through representation of community values and worldviews [22]. Similarities with research undertaken in Uganda and Kenya suggests that interventions may be transferrable across the wider region.

### Study limitations

Whilst a strength of this study was a comprehensive exploration of factors driving ABYM alcohol use through triangulation of several methods, including ethnographic observations, it has several limitations. First, the qualitative nature of the study, made it difficult to ascertain the scale of the problems described. Second, not all participants engaged in alcohol use and therefore reported on experiences and observations of other community members, rather than themselves. This might have led to under or over estimations. Finally, despite extensive efforts at rapport building some residual community suspicion about a possible link between the researchers and law enforcement agencies, may have led to social desirability of answers and underreporting of problems. To gain a more in-depth understanding of the proportion of adolescents that are affected by alcohol use and exposed to the drivers for alcohol use this study identified, future research should include a quantitative survey. area.

## Conclusion

Consumption of a range of types of alcohol, is common among ABYM in Northern Tanzania. This includes locally brewed (Mbege and Gongo) and commercially manufactured alcohol. Use is driven by a range of factors operating at multiple levels. Introduction to alcohol use in childhood due to cultural practices and/or misperceptions about the benefits of alcohol, high levels of alcohol availability in the home and community environments, and lack of regulatory enforcement are particularly important factors. Stressors such as expectations of financial independence combined with limited economic opportunities compound alcohol use as adolescence progresses. To be effective, preventive interventions will need to address this multiplicity of factors across different socio-ecological levels.

## Supporting information

S1 Appendix (DOCX)
