# Supplementary material for: “Alcohol will never run out”: Socio-ecological drivers of adolescent boys’ alcohol use in Kilimanjaro Region, Tanzania
Source: PLOS Glob Public Health. 2024 Jun 10;4(6):e0002443. doi: 10.1371/journal.pgph.0002443 (PMC11164390; doi:10.1371/journal.pgph.0002443)
Supplement: S1 Appendix — (DOCX) [file pgph.0002443.s001.docx]

**S1 Appendix**

|  | **Ubetu** | | **Njoro** | |
| --- | --- | --- | --- | --- |
| *In-depth interviews* | *Age* | *Sex* | *Age* | *Sex* |
| Community Health Worker | 70 | F | 27 | F |
| Community Health Worker | 44 | F | 26 | M |
| Out-of-school ABYM | 14 | M | 14 | M |
| Out-of-school ABYM | 14 | M | 14 | M |
| Out-of-school ABYM | 16 | M | 17 | M |
| Out-of-school ABYM | 14 | M |  |  |
| In-school ABYM | 18 | M | 17 | M |
| In-school ABYM |  |  | 14 | M |
| Religious leader | 26 | M | 27 | M |
| Alcohol brewer | Unknown | F | 65 | F |
| Alcohol brewer |  |  | 42 | F |
| Alcohol venue owner | 42 | M | 30 | F |
| Alcohol venue owner |  |  | 35 | M |
| Teacher | 33 | F | 42 | F |
| Teacher | 39 | M | 33 | M |
| Nurse | 53 | F | 58 | F |
| Community leader | 36 | F | 48 | F |
| Community leader |  |  | 50 | M |
| Salient person 1 | 68 | M | 33 | F |
| Salient person 2 | 42 | F | 72 | M |
| Salient person 3 |  |  | 62 | M |
| Salient person 4 |  |  | 58 | F |
|  |  |  |  |  |
| *Focus group discussions* | *N* | *Age range* | *N* | *Age range* |
| Out-of-school ABYM | 8 | 17-19 | 7 | 15-18 |
| In-school ABYM (old) | 6 | 15-17 | 6 | 15-16 |
| In-school ABYM (young) | 8 | 13-14 | 6 | 13-14 |
| Out-of-school AGYW | 6 | 18-19 | 8 | 17-24 |
| In-school AGYW | 8 | 16-18 | 5 | 15-16 |
| Mothers | 8 | 32-67 | 7 | 35-60 |
| Fathers | 7 | 38-65 | 6 | 38-64 |

**Age and occupation of Interview and FGD participants by study site**

NB: “Salient persons” were individuals who expressed salient perspectives during observations.
